# Structural Damage Detection through EMI and Wave Propagation Techniques Using Embedded PZT Smart Sensing Units

**DOI:** 10.3390/s22062296

**Published:** 2022-03-16

**Authors:** Himanshi Gayakwad, Jothi Saravanan Thiyagarajan

**Affiliations:** School of Infrastructure, Indian Institute of Technology Bhubaneswar, Argul, Khordha 752050, Odisha, India; 20se06010@iitbbs.ac.in

**Keywords:** piezoelectric sensor, impedance, embedded sensor, conductance, damage detection, COMSOL multiphysics, concrete

## Abstract

Lead Zirconate Titanate (PZT) sensors have become popular in structural health monitoring (SHM) using the electromechanical impedance (EMI) technique for damage identification. The vibrations generated during the casting process in concrete structures substantially impact the conductance signature’s (real part of admittance) magnitude and sensitivity. The concept of smart sensing units (SSU) is presented, composed of a PZT patch, an adhesive layer, and a steel plate. It is embedded in the concrete structure to study the impact of damage since it has high sensitivity to detect any structural changes, resulting in a high electrical conductance signature. The conductance signatures are obtained from the EMI technique at the damage state in the 10–500 kHz high-frequency range. The wave propagation technique proposes implementing the novel embedded SSUs to detect damage in the host structure. The numerical simulation is carried out with COMSOL multiphysics, and the received voltage signal is compared between the damaged and undamaged concrete beam with the applied actuation signal. A five-cycle sine burst modulated by a Hanning window is employed as the transient excitation signal. For numerical investigation, six cases are explored to better understand how the wave travels when a structural discontinuity is accounted for. The changes in the received signal during actuator–receiver mode in the damage state of the host structure are quantified using time of flight (TOF). Furthermore, the numerical studies are carried out by combining the EMI-WP technique, which implies synchronous activation of EMI-based measurements and wave stimulation. The fundamental idea is to implement EMI-WP to improve the effectiveness of SSU patches in detecting both near-field and far-field damage in structures. One SSU is used as an EMI admittance sensor for local damage identification. Meanwhile, the same EMI admittance sensor is used to acquire elastic waves generated by another SSU to monitor damages outside the EMI admittance sensor’s sensing area. Finally, the experimental validation is carried out to verify the proposed methodology. The results show that combining both techniques is an effective SHM method for detecting damage in concrete structures.

## 1. Introduction

Structural health monitoring (SHM) using smart piezoelectric materials is used for real-time, continuous, or automated monitoring of structures. Non-destructive testing (NDT) techniques such as impedance meters, the ultrasonic-based monitoring approach [1], the ultrasonic pulse velocity test [2], and the rebound hammer test [3] are examples of traditional SHM methods. NDT has several disadvantages, including being time-consuming, labor-intensive, needing large equipment, exposing inspectors to a potentially dangerous environment, and being inapplicable to crucial but inaccessible areas. These drawbacks are overcome by introducing smart piezoelectric materials, such as Lead Zirconate Titanate (PZT) transducer as its advantages include active sensing, wide frequency response, ability to respond quickly, low power consumption, low cost, and ease of installation.

**Figure 1:**
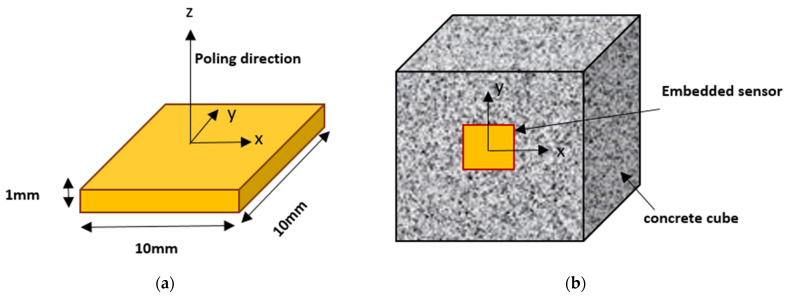
Schematic diagram: (**a**) PZT patch; (**b**) Concrete cube with embedded PZT transducer.

In recent years, there has been an increase in smart materials for structural monitoring. The electromechanical impedance (EMI) technique detects and localizes damage using the electromechanical coupling between the PZT patch and the host structure [4,5]. The PZT transducer is either bonded or embedded into the structure, and the schematic diagram of the concrete cube with an embedded sensor is shown in Figure 1. The embedded sensors for SHM gives the advantage of properly monitoring the internal changes in the concrete structure. Two piezoelectric-based monitoring techniques are the EMI-based and wave propagation (WP) techniques.

Several numerical and experimental studies have been conducted in concrete, steel, and aluminum structures to detect damage in the structures. Yang et al. [6] calculated the damage sensitivity and study on the PZT–steel and PZT–concrete structure interaction. Negi et al. [7] investigated different PZT sensor arrangements to identify the damage due to impact stress on the reinforced concrete slab. Cracks, degradation, and holes indicate possible structural damage. The signatures obtained in various stages are compared for damage detection to assess the changes that happened in the signatures [8,9,10]. The pioneering work of Shin et al. [11] monitored concrete hydration, whereas concrete strength prediction is studied by Soh et al. [12] in a detailed manner. The influence of damage and strength gain in EMI signature using a set of different embedded smart aggregates in a concrete cube are studied by Saravanan et al. [13,14].

The PZT sensor, adhesive layer, and steel plate are combined to form the novel sensing unit. The stiffness and mass of the sensor are changed by varying the thicknesses of the adhesive layer and the steel plate, such that the peak frequencies vary due to the different stiffness [15,16]. Sepehry et al. [17] used impedance-based SHM to evaluate the trend of strength development in an ancient structure with variable temperature, whereas Su et al. [18] looked at embedded piezoelectric sensors for measuring strength gain. Embedded PZT sensors are preferable over surface-bonded PZT, particularly as they help track interior concrete damages. The EMI signatures reveal a tendency to shift resonant peaks towards the right, and the amplitudes shift towards up [19,20]. The efficiency of the adhesive layer and the PZT sensor must be ensured for proper health monitoring; therefore, they must not be pre-damaged, as this could lead to misinterpretation of the results [21,22].

Damage detection via wave propagation is a transient dynamic phenomenon that aids in automated SHM [23,24]. The wave propagation approach uses two or more PZT transducers in which one transducer functions as an actuator and the others function as sensors, owing to the piezoelectric effect. They might be incorporated into the host structure or surface-bonded to it. Any kind of discontinuity or crack disrupted the traveling path of waves, which led to the study of damage in the structure using the wave propagation technique [25,26]. Using the PZT sensors in actuator–receiver mode, Lu et al. [27] and Luo et al. [28] explored how variations in the received stress wave effect damage evaluation. Ihn and Chang [29] developed vibration-based monitoring to detect damage in metallic structures. The experimental investigation on coupled axial–flexural wave propagation in a sagged rod with structural discontinuity using piezoelectric transducers is established [30,31]. Many researchers such as Krawczuk et al. [32], Gan et al. [33], Yang et al. [34], and Kumar et al. [35] have presented in their study that the presence of any discontinuity or crack disrupted the propagating wave. Damage monitoring, hydration monitoring, and early-strength monitoring in a concrete structure are further extended applications through wave propagation mechanics [36,37].

EMI and WP-based damage detection techniques have been widely used for SHM due to their sensitivity to small structural changes. Each of these techniques has its own technical merits, making them complementary. For example, the WP technique typically has a larger sensing range than the impedance technique, while the latter has better applicability to more complex structures. This paper aims to use EMI and wave propagation techniques to identify the structure’s damage precisely. The numerical investigations are carried out on the novel embedded SSUs to detect damage in the concrete structure. The analysis is carried out in COMSOL multiphysics [38], and the received signal is compared with the applied actuation signal between the damaged and undamaged concrete beams.

The present work combines the two methodologies, implying synchronized EMI-based measurements and wave propagation activation. The basic idea is to use EMI-WP to improve the effectiveness of the SSU patch in detecting both near-field and far-field damage in structures. This is accomplished by detecting variation in the admittance spectrum of the undamaged and damaged concrete beam by (a) activating only wave propagation technology and (b) activating an EMI admittance sensor SSU-2 at the same time when a wave generator at SSU-1 is activated. The findings demonstrate that combining the two techniques is an effective way to identify damages in concrete structures.

## 2. Mathematical Framework on EMI Technique

**Figure 2:**
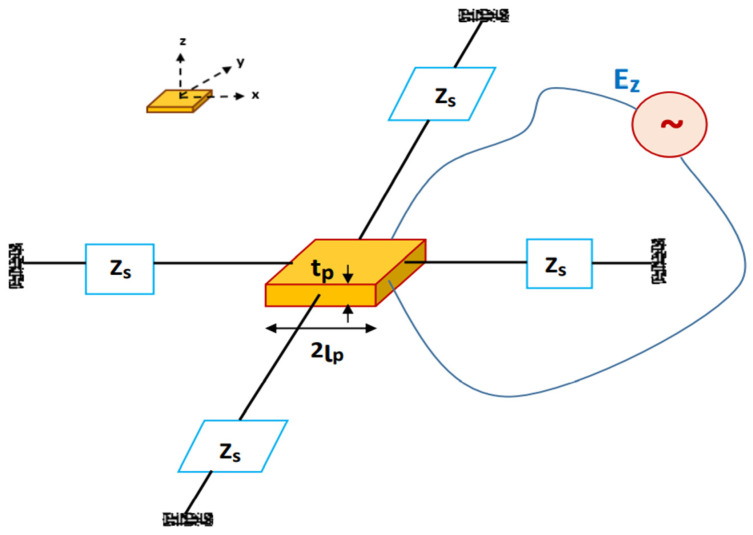
Interactive model of PZT patch and concrete structure.

EMI technique is utilized for monitoring the structural impedance response (*Z_s_*) from a bonded PZT sensor by supplying the voltage at a certain frequency to it. Figure 2 shows an interactive model of the PZT patch and concrete structure. The constitutive equations of a PZT patch, having a length of 2$l_{p}$ and a thickness $t_{p}$ [39,40] are expressed as:

(1) $$S_{x}=\frac{1}{{\bar{E}}_{p}}\left(T_{x}-\mu_{p}T_{y}\right)+d_{31}E$$

(2) $$S_{y}=\frac{1}{{\bar{E}}_{p}}\left(T_{y}-\mu_{p}T_{x}\right)+d_{31}E$$

(3) $$D={\overset{=}{\varepsilon }}_{33}E+d_{31}T_{x}+d_{32}T_{y}$$

where $\;S_{x}$ and $S_{y}$ are strains in the x and y directions, respectively; $\;T_{x}$ and $T_{y}$ are stresses in x and y direction, respectively; ${\bar{E}}_{p}=E_{p}(1+j\eta$) is the complex Young’s modulus of the PZT patch; η is the mechanical loss factor; $\;{\bar{\varepsilon }}_{33}^{T}=\varepsilon^{T}\left(1-j\delta \right)$ is the dielectric constant at zero stress; D is the electric displacement; δ is the dielectric loss factor; $d_{31}$ and $d_{32}$ are the piezoelectric constants in the x and y directions, respectively; and $\;\mu_{p}$ is Poisson’s ratio.

For isotropic material when $d_{31}=d_{32}$, the electric displacement D is written as:

(4) $$D={\bar{\varepsilon }}_{33}^{T}E+\frac{d_{31}{\bar{E}}_{p}}{1-\mu_{p}}\left(u^{\prime }+v^{\prime }-2d_{31}E\right)$$

where ()′ $=\frac{\partial ()}{\partial x}$, and u and  v are the displacement in x and y directions, respectively, whose solution is given as:

(5) $$\rho_{p}\ddot{u}=\frac{{\bar{E}}_{p}}{1-\mu_{p}^{2}}u^{\prime \prime }$$

(6) $$\rho_{p}\ddot{v}=\frac{{\bar{E}}_{p}}{1-\mu_{p}^{2}}v^{\prime \prime }$$

where (∙) $=\frac{\partial ()}{\partial t}$, and $\rho_{p}$ is the density of the PZT patch. The electric current flowing in the PZT sensor is expressed as:

(7) $$I=j\omega \int_{-\frac{l_{p}}{2}}^{\frac{l_{p}}{2}}\int_{-\frac{l_{p}}{2}}^{\frac{l_{p}}{2}}D\;dxdy$$

where $j=\sqrt{\left(-1\right)}$ is the complex number, and ω is the angular frequency. The electric field *E* is given as:

(8) $$E=\frac{V_{i}}{t_{p}}$$

where $V_{i}$ is the input voltage. The admittance is the ratio of current to the applied voltage. In the frequency domain, it is expressed as:

(9) $$Y\left(j\omega \right)=\frac{I\left(j\omega \right)}{V\left(j\omega \right)}$$

The admittance can be expressed in Fast Fourier Transform as:

(10) $$Y\left(j\omega \right)=\frac{FFT\;\left[I\left(t\right)\right]}{FFT\;\left[V\left(t\right)\right]}$$

Bhalla et al. [8] have used the effective impedance approach to formulate electromechanical admittance using an analytical model based upon an adhesively bonded PZT sensor. The equation for admittance signature is formulated using an analytical model and constitutive equations of the PZT patch expressed as:

(11) $$\bar{Y}=4\omega j\frac{{l_{p}}^{2}}{t_{p}}\left[{\bar{\varepsilon }}_{33}^{T}-\frac{2d_{31}^{2}\bar{Y^{E}}}{\left(1-\mu_{p}\right)}+\frac{2d_{31}^{2}\bar{Y^{E}}}{\left(1-\mu_{p}\right)}\left(\frac{Z_{a,eff}}{Z_{s,eff}+Z_{a,eff}}\right)\left(\frac{\tan\left(kl_{p}\right)}{\left(kl_{p}\right)}\right)\right]$$

where 2$l_{p}$ is the length PZT patch, *t*_*p*_ is the thickness of PZT patch, *ω* is the angular frequency, *d*_31_ is the piezoelectric strain coefficient, ${\bar{\varepsilon }}_{33}^{T}=\varepsilon^{T}\left(1-j\delta \right)$ is the complex electric permittivity of the PZT patch, $\;\bar{Y^{E}}=Y^{E}(1+j\eta$) is the complex Young’s modulus of the PZT patch,  η is the mechanical loss factor, *Z*_*a*__,__*eff*_ is the effective mechanical impedance of the PZT patch, *Z*_*s*__,__*eff*_ is the effective mechanical impedance of the host structure, $k=\omega \sqrt{\frac{\rho }{\bar{Y^{E}}}}$ is the wavenumber, ρ is the material density of the structure, and $\mu_{p}$ is the Poisson’s ratio of the PZT patch.

The electromechanical admittance *Y*(ω) is a complex quantity, expressed as:

(12) Y(ω)=G(ω)+jB(ω)

where G is the real part of admittance, known as conductance, and B is the imaginary part of admittance, known as susceptance. The frequency response functions, which carry crucial information about the structure’s health, are equivalent to the fluctuation in the PZT-EMI signature over a range of frequencies. The structural, mechanical impedance response *Z_s_* is the only PZT property that defines the overall admittance signature as all other PZT parameters are constant. As a result, any change in the EMI admittance signature signifies a change in structural integrity caused due to structural damage/discontinuity.

## 3. PZT Transducers for Understanding Wave Propagation Mechanics

The wave propagation technique uses two or more PZT transducers in which one of the PZT patches acts as an actuator, i.e., excited by a transient voltage (in the form of a sinusoidal tone burst). The converse piezoelectric effect produces a mechanical vibration due to the input voltage pattern. The bonding interface will transmit mechanical vibration to the host structure, which further changes into electrical signatures by the direct piezoelectric action when it reaches another patch, which acts as a sensor. PZT transducers can be attached to the surface of a structure or embedded into a concrete structure at a predetermined spacing for effective use. The change in impedance governs the ratio of a received signal to actuating wave, enabling the location and extent of the damage to be determined. As a result, wave propagation mechanics can be used to evaluate and detect damage [41,42,43,44,45].

The second-order partial differential equation governing wave propagation through a prismatic bar element is expressed as:

(13) $$c^{2}\frac{\partial^{2}u}{\partial x^{2}}-\frac{\partial^{2}u}{\partial x^{2}}=0$$

(14) $$c=\sqrt{\frac{E}{\rho }}$$

where *c* is the phase velocity in the axial direction, *u* is the displacement and *x* is the co-ordinate in the axial direction, *E* is Young’s modulus of elasticity, *A* is the area of bar element, and ρ is the density.

**Figure 3:**
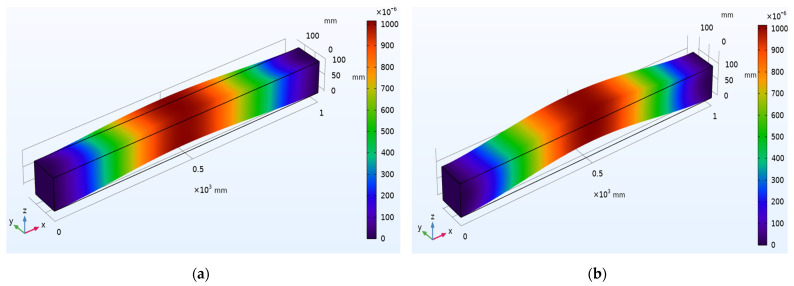
Surface displacement contours of beam for longitudinal mode shapes: (**a**) First mode; (**b**) Second mode.

**Figure 4:**
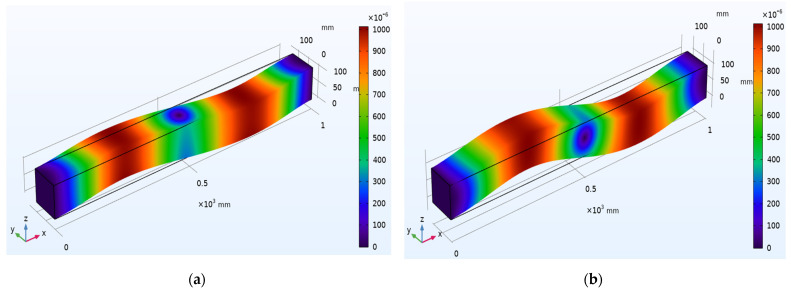
Surface displacement contours of beam for flexural mode shapes: (**a**) First mode; (**b**) Second mode.

**Figure 5:**
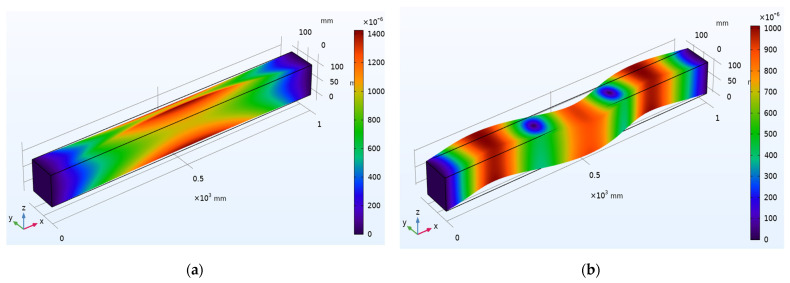
Surface displacement contours of beam for torsional mode shapes: (**a**) First mode; (**b**) Second mode.

Different propagation modes and dispersion patterns determine the wave motion through the waveguide. There are three fundamental modes: longitudinal, flexural, and torsional modes. A numerical simulation is carried out to understand the wave propagation mechanics using COMSOL™ Multiphysics 5.5 on a concrete beam with 1000 mm × 100 mm × 100 mm dimensions. Figure 3 depicts surface displacement contours of beam for the first and second longitudinal vibration modes with an Eigen frequency of 320.6 Hz. Similarly, Figure 4 represents the surface displacement contours of the beam for the first and second flexural modes of vibration with an Eigen frequency of 825.67 Hz. Figure 5 shows surface displacement contours of beam for the first and second torsional modes with Eigen frequencies of 980.72 Hz and 1499.5 Hz, respectively.

## 4. Numerical Simulations Using the EMI Technique

A numerical investigation is carried out to understand the impact of the damage through EMI response of the free-free PZT (before applying an epoxy coating) and embedded sensor in a concrete structure. These simulations are performed in COMSOL™ Multiphysics 5.5 using the piezoelectric effect under the module: structural mechanics, where a PZT-5A transducer is considered for the analysis. The material properties of PZT-5A are illustrated in Table 1.

The constitutive relations of the PZT material according to the naming convention used in COMSOL™ Multiphysics [38]:

(15) $$S=\frac{T}{C_{E}}+d^{T}E$$

(16) $$D=dT+\varepsilon_{T}E$$

where *S* is the strain vector of size (6 × 1), *T* is the stress vector of size (6 × 1) in Pa, $C_{E}$ is the elasticity matrix of size (6 × 6) in Pa, *E* is the electric field vector of size (3 × 1) in Volt/m, *D* is the electric displacement vector of size (3 × 1) in C/m^2^, $\;d^{T}$ is the piezoelectric coefficient of size (6 × 3) in m/Volt defined as the strain per unit field at constant stress, *d* is the electric displacement per unit stress at a constant electric field of size (3 × 6) in C/N, and $\varepsilon_{T}$ is the Dielectric permittivity of size (3 × 3) in Farad/m. The material properties of the epoxy layer are illustrated in Table 2.

**Figure 6:**
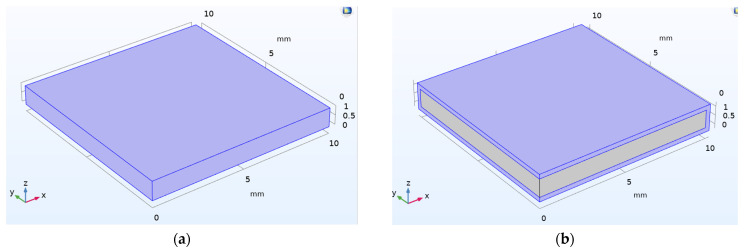

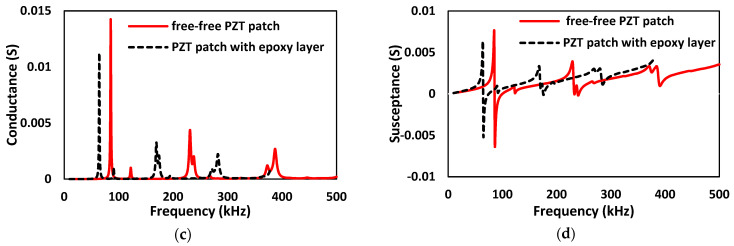
Representation of two-axis symmetry 3D model of (**a**) free-free PZT patch; (**b**) PZT patch with epoxy layer; (**c**) conductance and (**d**) susceptance plot based on the EMI measurement.

For numerical analysis, the three-dimensional (3D) modeling of the PZT sensor of the dimension 10 mm × 10 mm × 1 mm is considered where one of the 10 mm faces of the PZT sensor is used as a terminal with 1V applied voltage, while the opposite face is used as a ground as shown in Figure 6a. Figure 6b represents schematic two-axis symmetry 3D modeling of the PZT patch along with the epoxy coating. The dimension of the epoxy considered is 10.25 mm × 10.25 mm × 1.5 mm. The conductance signatures (real part of admittance) and susceptance signatures (imaginary part of admittance) of the free–free PZT patch and the PZT patch with an epoxy layer are obtained based on EMI measurement in the range of 10–500 kHz frequency at 1200 points, as shown in Figure 6c,d. It is inferred that the application of an epoxy coating surrounding the PZT patch results in a shift towards lower frequencies and a reduction in the peak conductance of the resonant peaks. The epoxy coating surrounding the PZT patch affects the resonant behavior. The material properties of concrete are illustrated in Table 3. The finite element (FE) mesh of the concrete beam is generated in COMSOL using a free tetrahedral mesh of finer element size considering maximum element size as 55 mm and minimum element size as 4 mm. The densification in the vicinity of PZT transducers is generated using a free tetrahedral mesh of extremely fine element size considering maximum element size as 20 mm and minimum element size as 0.2 mm.

**Figure 7:**
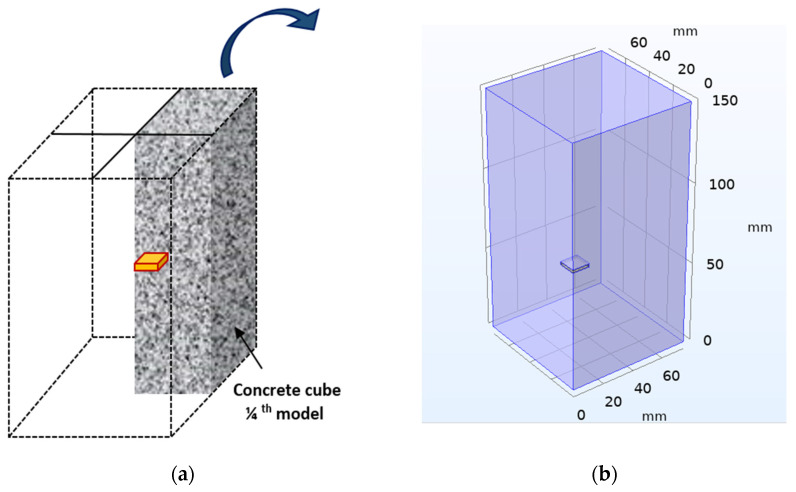
Concrete cube with embedded PZT sensor: (**a**) The schematic one-fourth model; (**b**) Two-axis symmetry 3D model.

**Figure 8:**
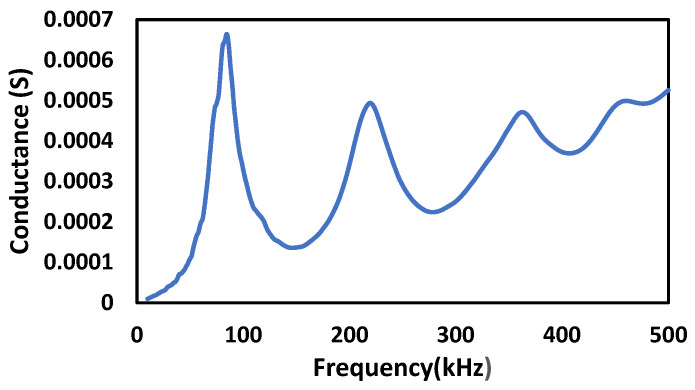
Conductance response obtained for embedded PZT patch in concrete.

Figure 7 shows a block representation of one-fourth model of the concrete cube with an embedded PZT sensor. A concrete cube of dimension 75 mm × 75 mm × 150 mm is modeled using a two-fold symmetry property. The same dimension of the PZT is considered, along with an epoxy coating of 1 mm thickness around the PZT patch. The EMI signature of embedded PZT patch was determined for the frequency ranges of 10 kHz to 500 kHz at 1200 point locations. Figure 8 represents the conductance signature obtained from EMI measurement when PZT patches are embedded in concrete.

## 5. Modeling of Smart Sensing Units

**Figure 9:**
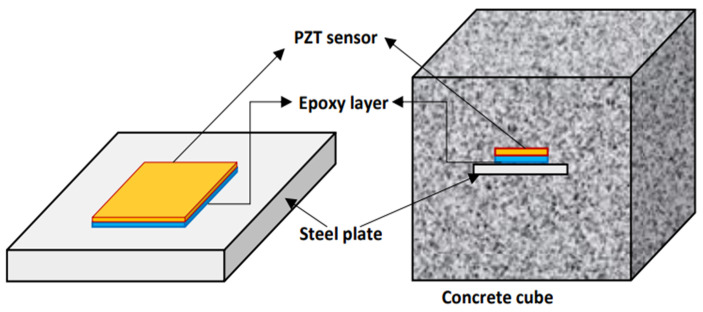
Schematic diagram concrete cube with embedded SSU.

**Figure 10:**
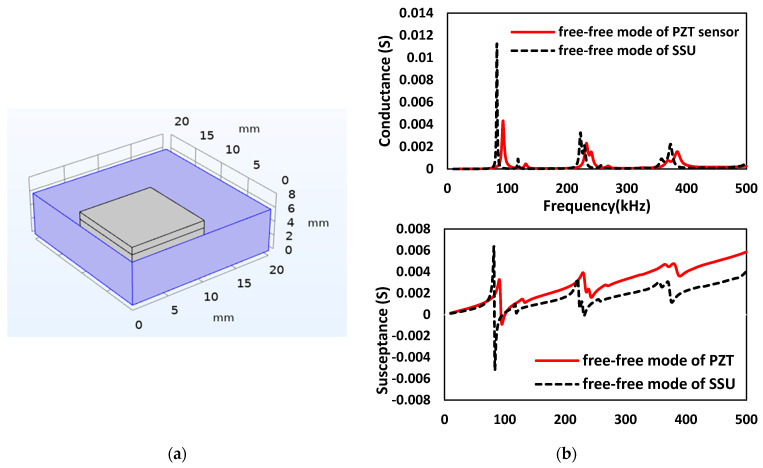
EMI response of SSU: (**a**) Schematic two-sided symmetry model of SSU; (**b**) Conductance and susceptance signatures obtained in a free–free mode of PZT sensors (without steel plate) and free–free mode SSU (with steel plate).

**Figure 11:**
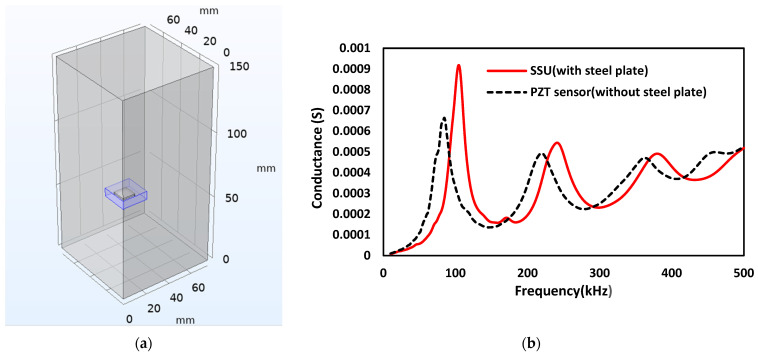
EMI measurement of SSU embedded in concrete: (**a**) One-fourth symmetry model; (**b**) Conductance response obtained.

In this section, the novel smart-sensing units (SSUs) are kept inside the concrete structures to examine the structural damage as they show high sensitivity to possible changes in the host structure. The SSU consists of a PZT sensor, adhesive layer, and steel plate, as shown in Figure 9. The steel plate of size 20 mm × 20 mm × 6 mm is used for the numerical investigation, as illustrated in Table 4. The sensor PZT-5A is considered with 10 mm × 10 mm × 1 mm acting both as actuator and sensor. Figure 10a shows the schematic two-sided symmetry 3D model of SSU, and Figure 10b represents the comparison conductance and susceptance spectrum between the free–free mode of PZT sensors (without steel plate) and the free–free mode of SSU (with steel plate). Figure 11a shows a block representation of the one-fourth model of SSU embedded in the concrete cube of dimension 75 mm × 75 mm × 150 mm, which is modeled using two-fold symmetry property. Figure 11b shows the EMI measurement response when the SSU is embedded in concrete. From the plot, it is inferred that the SSU embedded in concrete results in a shift towards higher frequencies and an increase in the peak conductance of the resonant peaks compared to the embedded PZT without a steel plate. As a result, it is concluded that SSU is more effective than the existing PZT sensors.

## 6. Numerical Simulations Using Wave Propagation Technique

**Figure 12:**
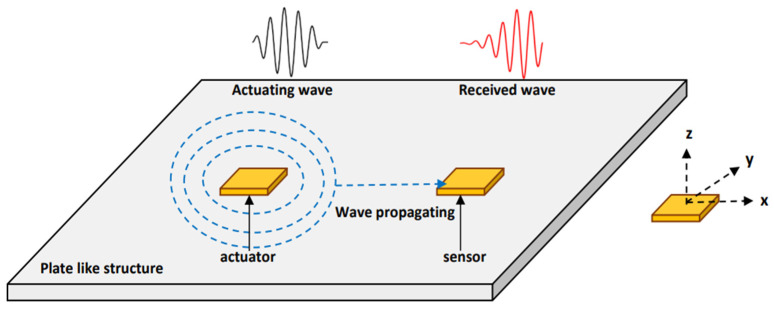
Schematic diagram of wave propagation with PZT transducer.

When two or more PZT transducers are considered in the analysis, one of the PZT patches acts as an actuator, i.e., excited by a transient voltage (in the form of sinusoidal tone burst) and the other acting as a receiver utilizing direct piezoelectric effect. PZTs are attached to the surface of a structure or embedded in the concrete structure at a predetermined spacing. A schematic diagram of wave propagation (WP) with a PZT transducer is shown in Figure 12. The multiphysics simulation model of WP with embedded SSUs in the concrete beam is constructed using the COMSOL™ Multiphysics computational platform. Two physical phenomena are included in the simulation model. First is the Solid mechanic’s module used to model the concrete beam, embedded SSUs, and epoxy layer. The second is electromechanical coupling, which involves simulating the piezoelectric effect of SSUs for wave excitation and sensing.

A concrete beam of dimension 1000 mm × 100 mm × 100 mm and a PZT patch of dimension 10 mm × 10 mm × 1 mm is considered for the analysis. The material properties of considered PZT-5A are illustrated in Table 1. For elastic waves generated by piezoelectric transducers, in the transient analysis, a five-cycle sine burst modulated by a Hanning window is given as actuating signal, expressed as:

(17) $$Y\left(t\right)=sin\left(2\pi ft\right)sin\left[\pi f\left(\frac{t}{N}\right)\right],\;\;0\leq t\leq \left(\frac{N}{f}\right)$$

where f is the frequency of the transducer, and N is the number of sine cycles.

In this section, the numerical investigations are performed considering six different cases, which are discussed below:

1. Undamaged concrete beam with surface-mounted PZT patches;
2. Damaged concrete beam with surface-mounted PZT patches;
3. Undamaged concrete beam with surface-mounted SSUs;
4. Damaged concrete beam with surface-mounted SSUs;
5. Undamaged concrete beam with embedded SSUs;
6. Damaged concrete beam with embedded SSUs.

FE simulation is performed for all the above cases. The discretization of the selected FE models is carefully allocated to assure the convergence to the proper solutions. Therefore, the considered FE models meet the commonly enforced accuracy standard of having at least seven FEs for the shortest wavelength inside the signal bandwidth of any waves that may travel in the waveguide [46]:

(18) $$\Delta_{x}\;\leq \frac{\lambda_{min}}{7}$$

(19) $$\Delta_{t}\;\leq 0.8\frac{\Delta_{x}}{V_{max}}$$

where $\lambda_{min}$ is the shortest wavelength, $\Delta_{x\;}$ is the mesh size of FE, $\Delta_{t}$ is the time taken by the wave to travel, and $V_{max}$ is the velocity of the fastest wave.

### 6.1. Case 1: Investigation on the Undamaged Concrete Beam with Surface-Mounted PZT Patches

**Figure 13:**
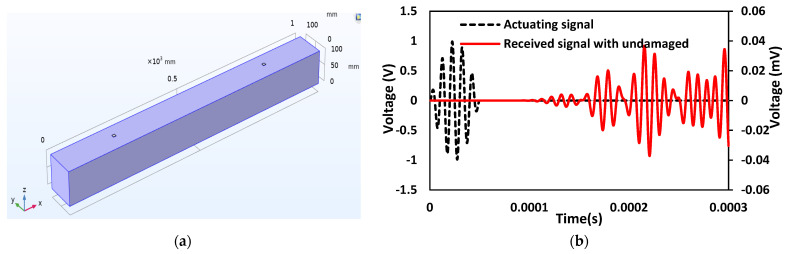
Concrete beam with two PZT sensors attached to the surface of the beam: (**a**) Schematic 3D model; (**b**) Response spectrum obtained from wave propagation measurements in the actuator–receiver mode.

First, the surface-mounted PZT is considered without any damage in the beam to obtain the response spectrum in the actuator–receiver mode. Figure 13a represents the schematic diagram of a concrete beam with two PZT sensors located 600 mm apart attached to the beam’s surface. PZT-1 and PZT-2 are 200 mm and 800 mm to the left of the beam, respectively. In this section, Rayleigh damping is used, with mass damping (α) equal to zero and stiffness damping (β) equal to 1.5 × 10^−8^. Figure 13b shows the transient analysis where five-cycle sine bursts are given as actuating signals at first PZT, and a second PZT acts as a receiver.

### 6.2. Case 2: Investigation on the Damaged Concrete Beam with Surface-Mounted PZT Patches

**Figure 14:**
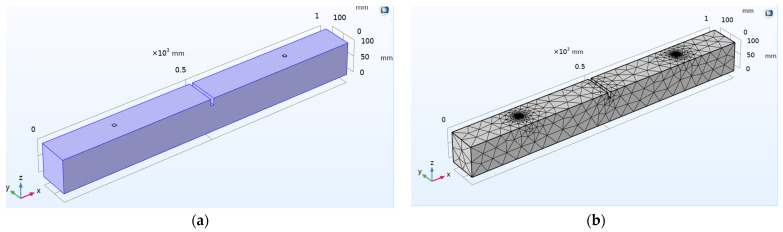

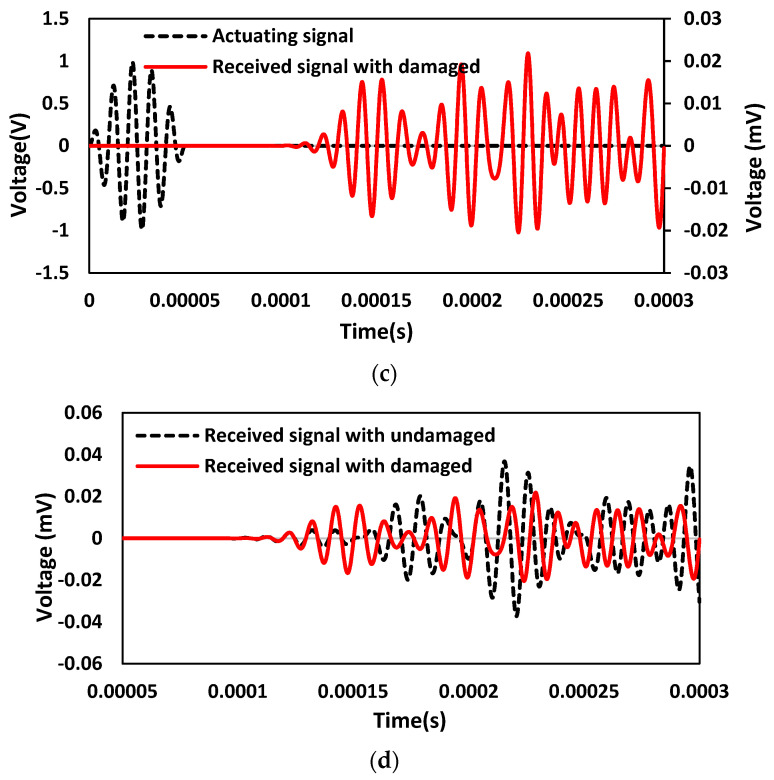
Damaged concrete beam with surface mounted PZT sensors: (**a**) Schematic 3D model; (**b**) FE model; (**c**) Response spectrum obtained from wave propagation measurements in the actuator–receiver mode; (**d**) Comparison of received signal between the damaged and undamaged concrete beam with the applied actuation signal.

Damage is introduced in the beam as structural discontinuity, and WP response is recorded in the time domain. Changes in the stiffness of certain elements in the mesh are used to introduce damages in this study. Figure 14a,b represent the schematic 3D view and FE model of the concrete beam, where damage is introduced as a crack in the beam. Figure 14c shows the actuating signal at first PZT and received voltage signal at second PZT in the damaged concrete beam. As shown in the plot, a crack in the transmission line causes a drop in the magnitude of the received voltage signal compared to the actuating voltage signal. The time of flight (TOF) is used to quantify the variation in the received voltage signal from actuating transient voltage in a wave measurement during the introduction of damage. Figure 14d compares received signals between the damaged and undamaged concrete beams. The observed decrease in the magnitude of the received voltage signal is due to a crack in the transmission path. Correspondingly, there is an increase in TOF with 0.0000261 s.

### 6.3. Case 3: Investigation on the Undamaged Concrete Beam with Surface-Mounted SSUs

**Figure 15:**
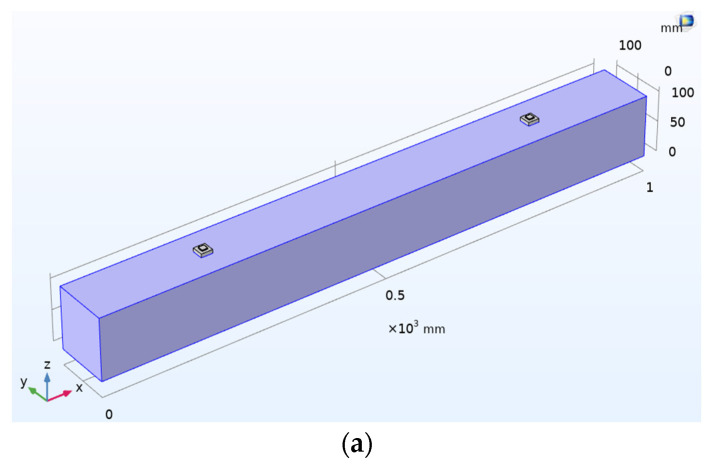

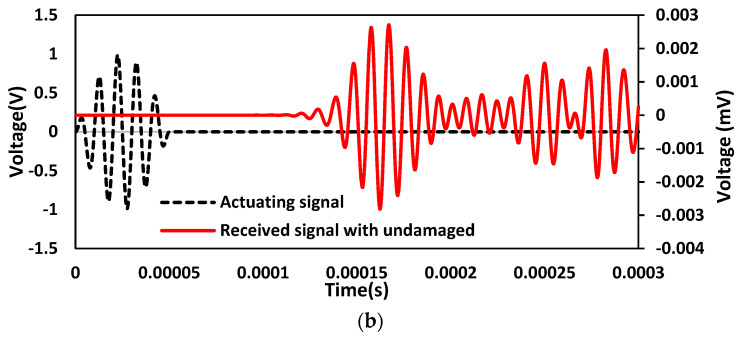
Concrete beam with surface-mounted SSU sensors: (**a**) Schematic 3D model; (**b**) Response spectrum obtained from wave propagation measurements in the actuator–receiver mode.

In this case, a surface-mounted SSU is considered to obtain the response spectrum in the actuator–receiver mode. The dimension considered for the concrete beam and the SSU is similar to the previous analysis. The material properties of the steel block are illustrated in Table 4. SSU-1 and SSU-2 are located at a distance of 200 mm and 800 mm, respectively, from the left side of the beam. Figure 15a represents the schematic 3D model of a concrete beam with two SSUs attached to the surface of the beam. Figure 15b shows the actuating signal at the first SSU and received signal at the second SSU.

### 6.4. Case 4: Investigation on the Damaged Concrete Beam with Surface-Mounted SSUs

**Figure 16:**
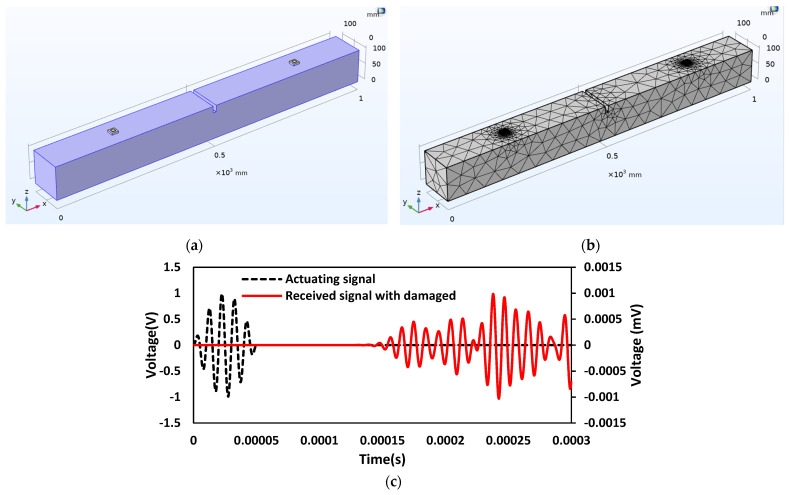

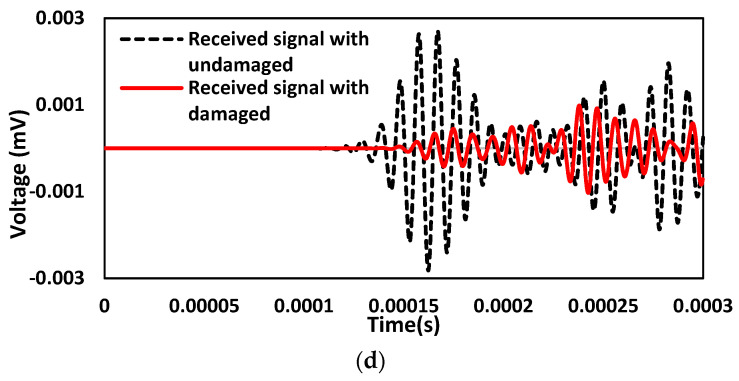
Damaged concrete beam with surface mounted SSUs: (**a**) Schematic 3D model; (**b**) FE model; (**c**) Response spectrum obtained from wave propagation measurements in the actuator–receiver mode; (**d**) Comparison of received signal between the damaged and no damaged concrete beam with the applied actuation signal.

Damage of 20 mm depth and 10 mm thickness is introduced at a location 300 mm away from SSU-1, and WP responses are recorded in the time domain. Figure 16a,b represent the schematic 3D model and FE model of the concrete beam, where damage is introduced as a crack in the beam. Figure 16c shows the actuating signal in a damaged concrete beam at SSU1 and received at SSU2. Figure 16d compares the received signal between the damaged and undamaged beam and the applied actuation signal when the SSU is surface mounted. The observed decrease in the magnitude of the received voltage signal is due to a crack in the transmission path. Correspondingly, there is an increase in TOF with 0.0000322 s.

### 6.5. Case 5: Investigation on the Undamaged Concrete Beam with Embedded SSUs

**Figure 17:**
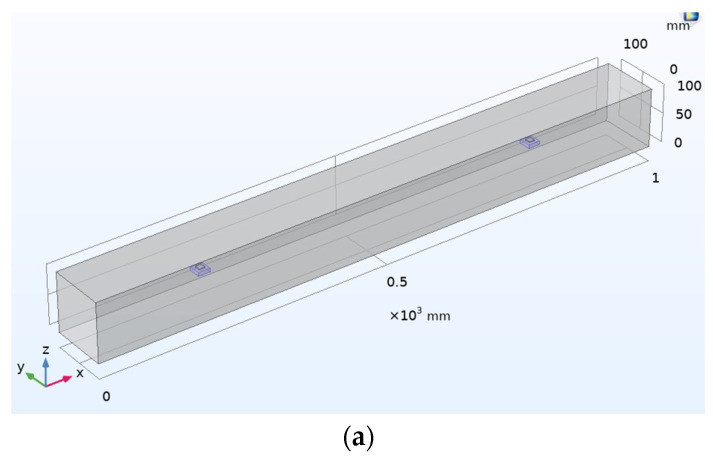

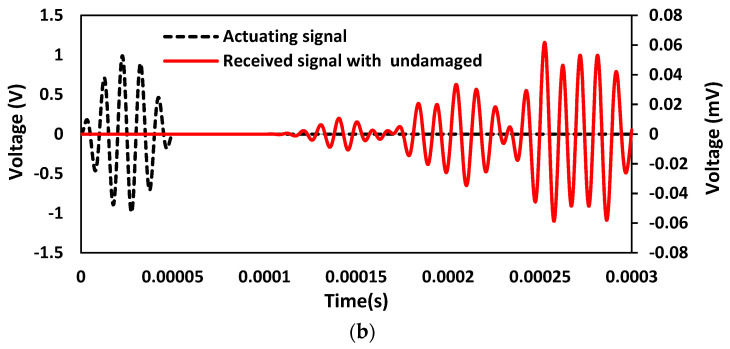
Concrete beam with embedded SSU: (**a**) Schematic 3-D model; (**b**) Response spectrum obtained from wave propagation measurements in the actuator–receiver mode.

WP measurement is performed on the embedded SSU in the concrete beam to obtain the time-dependent transmitted wave. In this analysis, one SSU is excited by a transient voltage acting as an actuator. The converse piezoelectric effect will produce a mechanical vibration that follows the voltage input pattern in the form of a five-cycle sine burst modulated by a Hanning window. The bonding interface will transmit mechanical vibration to the host structure, which further changes into electrical signatures by the direct piezoelectric action when it reaches another patch, which acts as a sensor. Figure 17a represents the schematic 3D model of a concrete beam with two SSUs embedded into the beam. Figure 17b shows the WP response of SSU in the form of actuated and received voltage signals.

### 6.6. Case 6: Investigation on the Damaged Concrete Beam with Embedded SSUs

**Figure 18:**
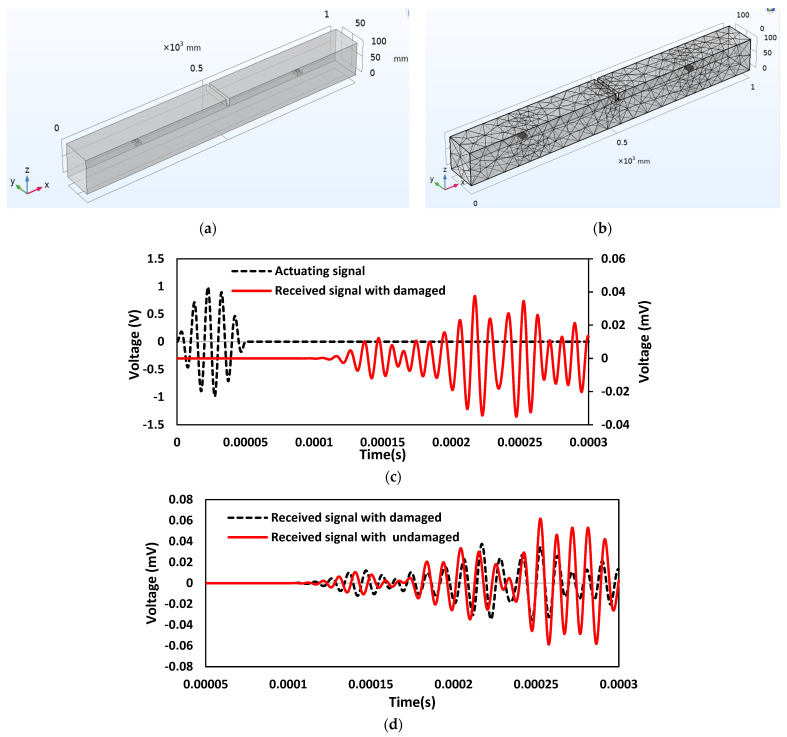
Damaged concrete beam with embedded SSU: (**a**) Schematic 3D model; (**b**) FE model; (**c**) Response spectrum obtained from wave propagation measurements in the actuator–receiver mode; (**d**) Comparison of received signal between the damaged and no damaged concrete beam with the applied actuation signal.

In this case, a structural discontinuity is introduced in the beam to study the changes occurring in transmitting waves, where damage is introduced as a crack with a depth of 20 mm and a thickness of 10 mm. The presence of damage along the direction of wave passage causes a change in the amplitude of the wave traveling. Figure 18a,b represent the schematic 3D and FE models of the damaged concrete beam, respectively. Figure 18c shows the wave propagation response of embedded SSU in actuator–receiver mode. The comparison plot of the received signal between the damaged and undamaged concrete beam with the applied actuation signal using embedded SSU is shown in Figure 18d. The variation of the received signal indicates the presence of a crack in the path of a wave traveling. Correspondingly, there is an increase in TOF with 0.0000155 s.

**Figure 19:**
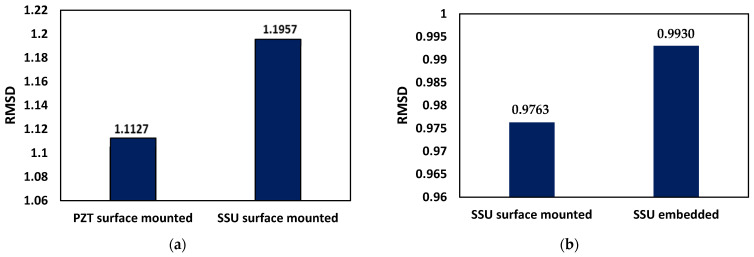
RMSD comparison plot for a damaged concrete beam with: (**a**) surface-mounted PZT patches (case-2) and SSUs (case-4); (**b**) surface-mounted SSUs (case-4) and embedded SSUs (case-6).

The statistical metric to quantify the changes in the waveform is employed using root mean square deviation (RMSD) expressed as:

(20) $$\mathrm{RMSD}=\sqrt{\frac{\sum {\left(x_{i}^{o}-x_{i}^{1}\right)}^{2}}{\sum {\left(x_{i}^{o}\right)}^{2}}}$$

where $x_{i}^{o}$ is the *i*th value of reference signature, and $x_{i}^{1}$ is the *i*th value of the compared signature. Figure 19a shows the RMSD plot obtained from the received voltage signal for case-2 (*damaged concrete beam with surface-mounted PZT patches*) and case-4 (*damaged concrete beam with surface-mounted SSUs*), using the respective undamaged cases as a reference. It is feasible to conclude that utilizing WP response with SSU improves the RMSD value for damage detection, indicating that SSUs are more effective than smart PZT sensors. Similarly, the RMSD plot is obtained for case-4 (*damaged concrete beam with surface-mounted SSUs*) and case-6 (*damaged concrete beam with embedded SSUs*) with case-2 (*damaged concrete beam with surface-mounted PZT patches*) as reference data. Figure 19b concludes that employing embedded SSUs for damage detection in concrete structures can efficiently monitor interior damage through WP response.

## 7. Combining EMI and Wave Propagation Techniques for Robust Damage Detection

**Figure 20:**
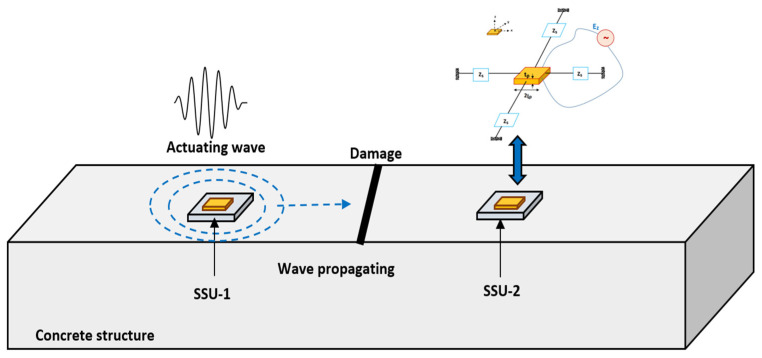
Schematic representation of the combined EMI-WP technique for damage detection in concrete structure utilizing two SSUs.

The performance and reliability of damage detection are enhanced using impedance and guided wave signals simultaneously obtained from PZTs attached to a target structure [47]. The proposed technique takes advantage of the EMI and WP-based damage detection techniques and can effectively detect damage in structures. Combining the EMI-WP technique implies synchronous activation of EMI-based measurements and wave stimulation. The fundamental idea is to implement EMI-WP to improve the effectiveness of SSU patches in detecting both near-field and far-field damage in structures. In this investigation, at least two similar types of SSU are used for EMI response and wave propagation in actuator–receiver mode. One SSU is used as an EMI admittance sensor for local damage identification. Meanwhile, the same EMI admittance sensor is used to acquire elastic waves generated by another SSU to monitor damages outside the EMI admittance sensor’s sensing area, as shown in Figure 20.

**Figure 21:**
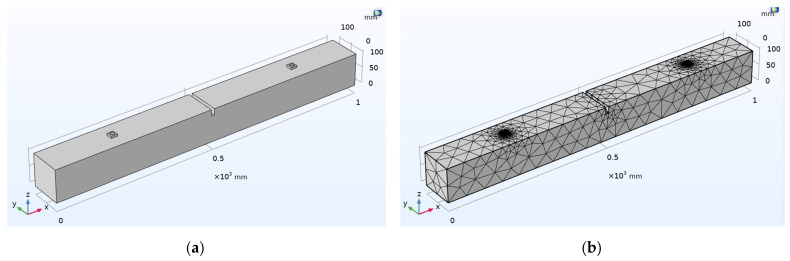
Combined EMI-WP measurement: (**a**) 3D model; (**b**) FE model of a damaged concrete beam with surface-mounted SSUs.

A numerical investigation is carried out in a concrete beam with a rectangular cross-section of 100 mm × 100 mm and a length of 1000 mm, as shown in Figure 21. The concrete beam has two SSU surfaces attached to it. The material properties of PZT-5A are shown in Table 1, and the properties of steel blocks are shown in Table 4. SSU-1 and SSU-2 are located at a distance of 200 mm and 800 mm, respectively, from the left side of the beam. At a distance of 300 mm from SSU-1, damage with a depth of 20 mm and a thickness of 10 mm is introduced. As illustrated in Figure 20, SSU-2 is activated to acquire EMI signature for localized damage detection, while the same SSU-2 is being used to sense wave propagating from SSU-1, accounting for far-field damage identification. SSU-1 is activated by a five-cycle sine burst transient excitation signal modulated by a Hanning window.

Four different conditions are investigated:

(A) Active wave propagation in an undamaged concrete beam;
(B) Active wave propagation in damaged concrete beam;
(C) EMI admittance sensor SSU-2 is activated simultaneously with a wave generator at SSU-1 in an undamaged concrete beam;
(D) EMI admittance sensor SSU-2 is activated simultaneously with a wave generator at SSU-1 in a damaged concrete beam.

**Figure 22:**
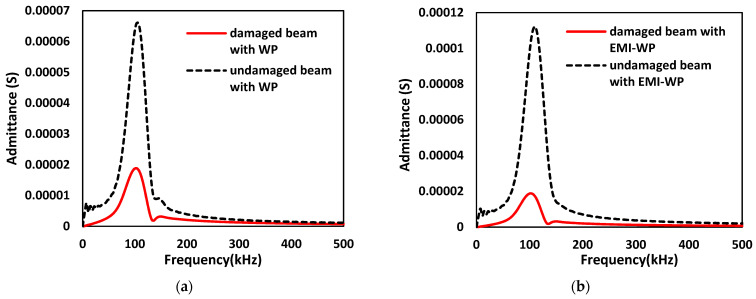
FFT Admittance spectrums for an undamaged and damaged concrete beam with: (**a**) Active wave propagation; (**b**) When EMI admittance sensor is activated simultaneously with wave propagation.

The comparison of the Fast Fourier Transform (FFT) admittance spectrum for an undamaged and damaged concrete beam with active wave propagation is shown in Figure 22a. The results are based on a 100kHz excitation frequency. It is the result for cases (A) and (B). A variation in the admittance spectrum indicates the damage in the structure. Figure 22b shows the FFT admittance spectrum for undamaged and damaged cases when EMI admittance sensor SSU-2 is activated simultaneously with a wave generator at SSU-1, resulting from cases (C) and (D).

**Figure 23:**
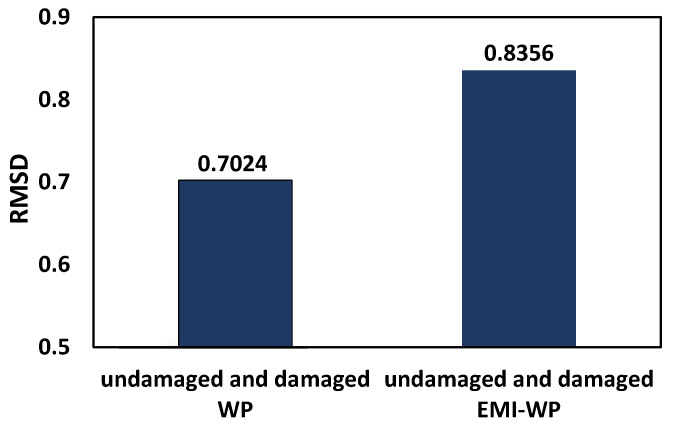
RMSD plot for a concrete beam under different structural conditions.

From the plot, it is observed that the sensing capabilities of SSUs are enhanced when EMI-WP techniques are simultaneously enabled, which helps in better damage detection. The RMSD index of the absolute value of the admittance spectrum calculated under the investigated four distinct conditions is shown in Figure 23, using the undamaged case as a reference. As an outcome, it is concluded that combining the EMI measurement with wave propagation enhances the RMSD value from 0.7024 to 0.8356. Finally, improved damage diagnosis is performed using both impedance and wave signals.

## 8. Experimental Validation

**Figure 24:**
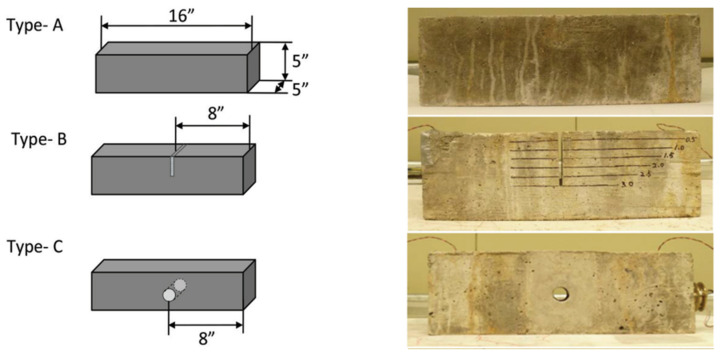
Experimental specimen with crack and hole [48].

**Figure 25:**
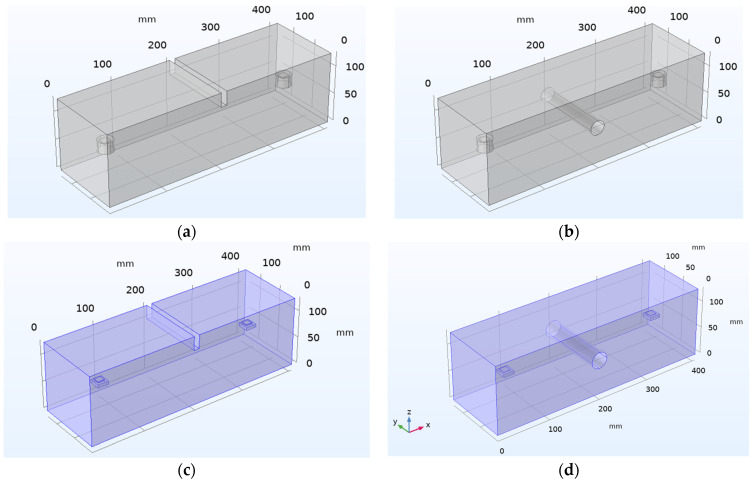
Three-dimensional model geometry: (**a**) Type-B specimen as crack damage; (**b**) Type-C specimen as hole damage; (**c**) Type-B specimen as crack damage with embedded SSUs; (**d**) Type-C specimen as hole damage with embedded SSUs.

The experimental study is performed on the concrete beam with embedded PZT patches to detect damage using wave propagation technology [48]. The test specimen is shown in Figure 24. Two embedded PZTs are employed for this purpose, one functioning as an actuator to generate the wave and the other as a sensor. The smart aggregate model contains a concrete cylinder and a PZT patch. Small concrete cylinders having a diameter of 25.4 mm and a depth of three-fourths of 25.4 mm are used, with PZT patches of 12.7 mm × 12.7 mm × 1 mm embedded at the two ends of each specimen on the central axis. A concrete beam of a 127 mm × 127 mm rectangular cross-section and a length of 406.4 mm is modeled. Damage is simulated as a notch in the center of the concrete beam with varying depths of 25.4, 50.8, 76.2, and 101.6 mm, and as a hole in the middle of the concrete beam with different diameters of 25.4, 38.1, 50.8, and 63.5 mm is shown in Figure 25.

**Figure 26:**
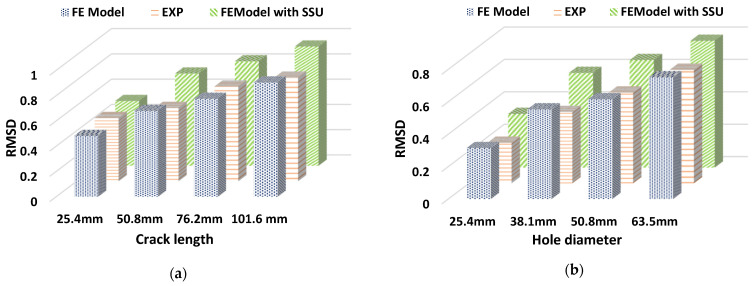
RMSD comparison plot between numerical simulation of existing smart aggregate, experimental results, and numerical simulation with SSU: (**a**) for a beam with crack; (**b**) for a beam with hole.

For wave generated by piezoelectric transducers, in the transient analysis, a 3.5 cycle tone burst modulated by a Hanning window is given as actuating signal, expressed as:

(21) $$Y\left(t\right)=\left[1-cos\left(\frac{2\pi f}{N}t\right)\right]sin\left(2\pi ft\right),\;0\leq t\leq \left(\frac{N}{f}\right)$$

where f= 100 kHz is the frequency of the transducer, and N= 3.5 cycles is the number of cycles used in the analysis. Figure 26 shows the RMSD plot for different crack depths and hole diameters in the concrete beam acquired in the experimental analysis and numerical simulation, with the undamaged cases as a reference. The plot shows that an increase in the crack depth causes a decrease in the magnitude of the received signal, which is expressed quantitatively using the RMSD damage index. The plot shows that the numerical simulation and experimental results are well-matched.

Likewise, for a damaged concrete beam for different crack depths and hole diameters with embedded SSUs, the RMSD value increases. The figure shows that using embedded SSUs for damage detection in concrete structures may effectively monitor damage through WP response, making embedded SSUs a more effective modeling technique for damage identification than existing smart aggregate models. Combining the EMI-WP technique implies synchronized activation of EMI-based measurements and wave propagation in the proposed technique for both near-field and far-field damage detection. A numerical investigation is carried out in a concrete beam of dimension 406.4 mm × 127 mm × 127 mm with two embedded SSUs as smart aggregates towards the two ends of each specimen on the central axis, as shown in Figure 25c,d. Two damage cases are introduced as a crack of depth 25.4 mm and hole of diameter 38.1 mm, and a thickness of 10 mm in the center, respectively.

**Figure 27:**
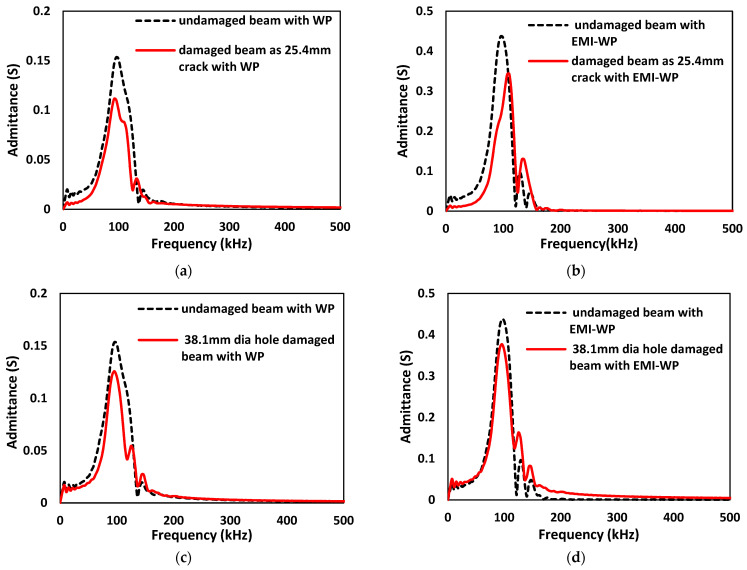
FFT Admittance spectrums for an undamaged and damaged concrete beam with: (**a**,**c**) active wave propagation; (**b**,**d**) when an EMI admittance sensor is activated simultaneously with wave propagation.

**Figure 28:**
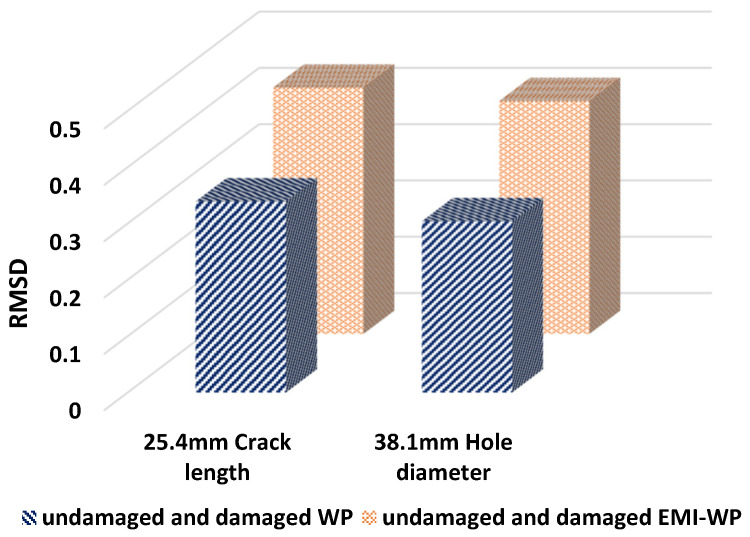
RMSD plot for a concrete beam under different structural conditions.

Four different conditions are investigated, as explained in Section 7. The FFT admittance spectrum is obtained for an undamaged and damaged concrete beam with active wave propagation as shown in Figure 27a for the crack length of 25.4 mm and Figure 27c for a hole diameter of 38.1 mm. These cases are similar to cases (A) and (B) in Section 7. The variation is observed in the admittance spectrum due to damage in the structure. Figure 27b,d show the FFT admittance spectrum for undamaged and damaged cases for the crack length of 25.4 mm and for a hole diameter of 38.1 mm, respectively, when EMI admittance sensor SSU-2 is activated simultaneously with a wave generator at SSU-1, resulting from cases (C) and (D). The RMSD index of the absolute value of the admittance spectrum calculated under the investigated four distinct conditions is shown in Figure 28, using the undamaged case as a reference.

The sensing capabilities of SSUs are improved when EMI-WP techniques are combined, and the RMSD value is increased, resulting in greater damage identification. As a result, the embedded SSU is recommended as a more effective modeling technique for damage identification than the existing smart aggregate models.

## 9. Conclusions

This paper explains how to use EMI and WP to identify damage in the concrete structure. The WP technique is utilized since EMI measurements cannot identify damage outside the PZT sensor’s detection range. The following conclusions are derived from the study:

- WP-based measurement shows that damage in the structure is successfully detected utilizing the embedded SSU by observing the variation in magnitude of the received voltage signal and improved RMSD value.
- Comparing the WP response in the actuator-sensor mode of the embedded SSU, it is observed that the decrease in the magnitude of the received voltage signal is because of a crack in the transmission path, and the increase in the time of flight is due to the presence of crack along the path of the wave propagating.
- The numerical value of the RMSD employing an SSU instead of PZT patches for damage detection exhibited by six separate cases in the wave propagation technique showed that steel–PZT models are preferable to smart material PZT-based models.
- EMI measurements are particularly important because they can detect damage to the embedded PZT patch or debond between the epoxy and concrete interface. Therefore, combining EMI and WP measurements for damage identification is useful for detecting relatively close and far damages in the structures.
- The results of the combined EMI-WP approach using four structural conditions show that the presence of damage is indicated by a change in the FFT admittance spectrum, as well as by the increment in the RMSD value from a damaged concrete beam where only wave propagation is active to EMI admittance sensor SSU-2 being activated simultaneously.
- The numerical results are compared with experimental analysis for different crack lengths and holes, which shows a good fit between the 3D FE model results and experimental results.
- The FE model results are compared with the experimentally available smart aggregate and proposed embedded SSU for damage detection, displaying enhanced damage index value. Thus, it is recommended that the embedded SSU results in a more effective modeling technique than the existing smart aggregate models for damage detection.
- The combined EMI-WP-based robust damage detection is developed by utilizing impedance and wave signals simultaneously obtained from SSUs to enhance the performance and reliability of damage diagnosis.

## Figures and Tables

**Table 1:** Material properties of PZT patch.

Parameters	Value
Density, *ρ* [kg/m^3^]	7750
Poisson’s ratio, v	0.3
Damping ratio, ζ	0.012
Dielectric loss factor, δ	0.0169
Elasticity matrix [Pa]	$\left[\begin{array}{cccccc} 1.20\times 10^{11} & 7.51\times 10^{10} & 7.50\times 10^{10} & 0 & 0 & 0\\ 7.51\times 10^{10} & 1.20\times 10^{11} & 7.50\times 10^{10} & 0 & 0 & 0\\ 7.50\times 10^{10} & 7.50\times 10^{10} & 1.10\times 10^{11} & 0 & 0 & 0\\ 0 & 0 & 0 & 2.10\times 10^{10} & 0 & 0\\ 0 & 0 & 0 & 0 & 2.10\times 10^{10} & 0\\ 0 & 0 & 0 & 0 & 0 & 2.25\times 10^{10} \end{array}\right]$
Piezoelectric Constants [C/N]	$\left[\begin{array}{cccccc} 0 & 0 & 0 & 0 & 5.84\times 10^{-10} & 0\\ 0 & 0 & 0 & 5.84\times 10^{-10} & 0 & 0\\ -1.71\times 10^{-10} & -1.71\times 10^{-10} & 3.74\times 10^{-10} & 0 & 0 & 0 \end{array}\right]$
Relative permittivity	$\left[\begin{array}{ccc} 1730 & 0 & 0\\ 0 & 1730 & 0\\ 0 & 0 & 1700 \end{array}\right]$

**Table 2:** Properties of the Epoxy layer.

Parameters	Variable	Value
Young’s modulus	E	2 GPa
Density	ρ	1250 kg/m^3^
Poisson’s ratio	v	0.4

**Table 3:** Properties of concrete.

Parameters	Variable	Value
Young’s modulus	E	25 GPa
Density	ρ	2300 kg/m^3^
Poisson’s ratio	v	0.20

**Table 4:** Properties of the steel plate [38].

Parameters	Variable	Value
Young’s modulus	E	200 GPa
Density	ρ	7850 kg/m^3^
Poisson’s ratio	v	0.30

## Data Availability

Not applicable.
